# An agenda-setting paper on data sharing platforms: euCanSHare workshop

**DOI:** 10.12688/openreseurope.13860.1

**Published:** 2021-07-19

**Authors:** Thijs Devriendt, Clemens Ammann, Folkert W. Asselbergs, Alexander Bernier, Rodrigo Costas, Matthias G. Friedrich, Josep L. Gelpi, Marjo-Riitta Jarvelin, Kari Kuulasmaa, Karim Lekadir, Michaela Th. Mayrhofer, Vaclav Papez, Gerard Pasterkamp, Steffen E. Petersen, Carsten Oliver Schmidt, Jeanette Schulz-Menger, Stefan Söderberg, Mahsa Shabani, Giovanni Veronesi, Darian Steven Viezzer, Pascal Borry

**Affiliations:** 1 Centre for Biomedical Ethics and Law, Department of Public Health and Primary Care, KU Leuven, Leuven, Belgium; 2 Working Group on Cardiovascular Magnetic Resonance, Experimental and Clinical Research Center, a joint cooperation between the Charité - Universitätsmedizin Berlin and the Max-Delbrück-Center for Molecular Medicine, Berlin, Germany; 3 Department of Cardiology, Division Heart & Lungs, University Medical Center Utrecht, Utrecht University, Utrecht, The Netherlands; 4 4Institute of Cardiovascular Science, Faculty of Population Health Sciences, University College London, London, UK; 5 Institute of Health Informatics, University College London, London, UK; 6 Health Data Research UK, London, UK; 7 Centre of Genomics and Policy, Faculty of Medicine, McGill University, Montreal, Canada; 8 Centre for Science and Technology Studies (CWTS), Leiden University, Leiden, The Netherlands; 9 Departments of Medicine and Diagnostic Radiology, McGill University Health Centre, Montreal, Canada; 10 Department of Biochemistry and Molecular Biomedicine, University of Barcelona, Barcelona, Spain; 11 Barcelona Supercomputing Center (BSC), Barcelona, Spain; 12 Department of Life Sciences, College of Health and Life Sciences, Brunel University London, London, UK; 13 MRC-PHE Centre for Environment and Health, Department of Epidemiology and Biostatistics, School of Public Health, Imperial College London, London, UK; 14 Center for Life Course Health Research, Faculty of Medicine, University of Oulu, Oulu, Finland; 15 Unit of Primary Health Care, Oulu University Hospital, Oulu, Finland; 16 National Institute for Health and Welfare, Helsinki, Finland; 17 Artificial Intelligence in Medicine Lab (BCN-AIM), Department of Mathematics and Computer Science, University of Barcelona, Barcelona, Spain; 18 BBMRI, Graz, Austria; 19 Department of Clinical Diagnostics Laboratories, University Medical Center Utrecht, Utrecht University, Utrecht, The Netherlands; 20 Barts Heart Centre, Barts Health NHS Trust, London, UK; 21 William Harvey Research Institute, NIHR Barts Biomedical Research Centre, Queen Mary University of London, London, UK; 22 The Alan Turing Institute, London, UK; 23 Institute for Community Medicine, Department SHIP-KEF, Greifswald University Medical Center, Greifswald, Germany; 24 DZHK (German Centre for Cardiovascular Research) partner site, Berlin, Germany; 25 Department of Cardiology and Nephrology, HELIOS Hospital Berlin-Buch, Berlin, Germany; 26 Department of Public Health and Clinical Medicine, Heart Centre, Umeå University, Umeå, Sweden; 27 METAMEDICA, Department of Law and Criminology, Ghent University, Ghent, Belgium; 28 Research Center in Epidemiology and Preventive Medicine (EPIMED), Department of Medicine and Surgery, University of Insubria in Varese, Varese, Italy

**Keywords:** data sharing, data infrastructure, open science, incentives, science policy

## Abstract

Various data sharing platforms are being developed to enhance the sharing of cohort data by addressing the fragmented state of data storage and access systems. However, policy challenges in several domains remain unresolved. The euCanSHare workshop was organized to identify and discuss these challenges and to set the future research agenda. Concerns over the multiplicity and long-term sustainability of platforms, lack of resources, access of commercial parties to medical data, credit and recognition mechanisms in academia and the organization of data access committees are outlined. Within these areas, solutions need to be devised to ensure an optimal functioning of platforms.

## Plain language summary

Public funds are being spent on infrastructures that can help the sharing of medical data. However, to make these infrastructures work optimally, we need to overcome barriers to data sharing. In addition, we need to make sure that platforms themselves function well. This means that we need to think about potential overlap between platforms. We also need to make sure that platforms have enough public funds to keep running. Furthermore, the researchers that have medical data should have sufficient funds to prepare the data before it is contributed to platforms. We also need to make sure that researchers that share medical data are recognized and rewarded. Lastly, we need to organize committees that decide on data access so that doubled work is limited where possible. If all these factors are addressed, we can make sure that infrastructures are sustainable in the long-term and that they can best serve science. 

## Introduction

The integrated analysis of detailed social, environmental and lifestyle factors, and genetic determinants is critical to a better understanding of diseases, especially the risk and pathophysiology of complex conditions, with the goal of benefitting the patient or the general population ^1^. To achieve this, extensive efforts are undertaken to harmonize heterogeneous datasets for cross-study comparisons and informative meta-analysis, in particular in the field of population health studies ^2– 4^. Despite the obvious advantages of such harmonized and integrated datasets, multiple barriers exist that can stymie these efforts. Poor data quality, fear of misinterpretation of data and the resulting reputational harm, a lack of resources to prepare data for sharing, as well as ethical and legal restrictions, and a lack of incentives have been reported as reasons for not sharing data ^5– 7^. Medical data also remain fragmented across numerous medical institutions and sometimes even within the same institution across units, including research hospitals and universities, each featuring their own data access conditions, privileges, and procedures. Although these local systems may be designed to efficiently request and provide access to data, they are generally not aimed at facilitating external data integration and reuse. This is especially the case when their primary aim is clinical care and research is understood as secondary in contrast to being complementary. The plentitude of different data management systems and data models, data standards and phenotype definitions therefore complicates the interinstitutional exchange of data and the data’s integration for individual research projects. From this perspective, medical data most often cannot be considered as FAIR (findable, accessible, interoperable and reusable) ^8^. The accessibility criterion is generally understood to mean “accessible under well-defined conditions”, as restrictions to access for sensitive medical data occur under the General Data Protection Regulation (GDPR) ^8– 11^. As academic research is mostly funded with taxpayer money, the lack of FAIR data diminishes the return on investment for the public. This is also accompanied by an economic loss, with one report by PricewaterhouseCoopers, LLP (PwC) EU Services estimating that the quantifiable, measurable cost of not having FAIR research data is 10.2 billion euros per year ^12^. To address this situation, the European Commission is funding the development of various data sharing platforms (hereafter “platforms”) for cohort data. These platforms generally aim to: (a) develop data catalogues to increase the visibility of research studies and provide an overview of shared and harmonized variables across cohorts; (b) introduce data access procedures in which consent criteria are represented by standardized Data Use Ontology (DUO) codes and data requests are matched to these codes semi-automatically; (c) allow the storage of cohort data in local or specialized repositories, which are mapped into a single federated network; and (d) provide cloud-based virtual research environments that enable federated analyses of data residing in local repositories.

**Figure 1.**
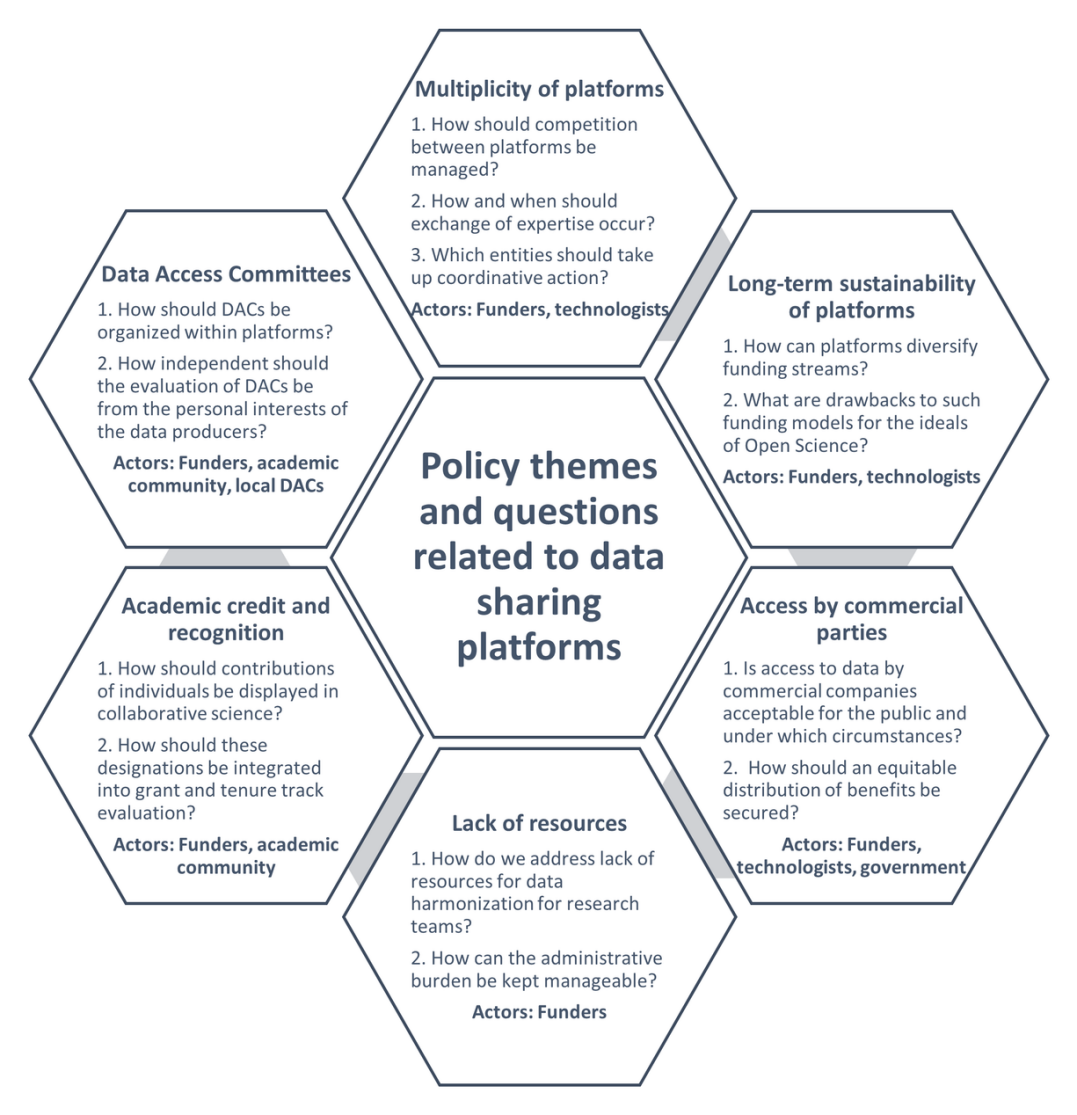
Key questions on each policy theme.

Although efforts to establish such platforms across Europe and Canada are ongoing, these initiatives may face various organizational and governance challenges. Salient questions emerge in multiple policy domains that require appropriate answers if platforms are to operate optimally. One such data sharing platform focusing on cardiovascular and imaging data is being built by the euCanSHare consortium (an EU- Canada joint infrastructure for next-generation multi- Study Heart research). As part of the project, the “euCanSHare Workshop on Incentives for Data Sharing” was set up to discuss policy challenges for platforms. The workshop convened on the 29 and 30 ^th^ of September 2020 in an online session which brought together members of euCanSHare with other experts from various disciplines, such as medical professionals, cardiovascular researchers, bioethics and social science experts, and data technologists. The primary objective was to discuss the barriers, challenges and opportunities for data sharing in cardiovascular research as data sharing platforms become more established. During the meeting, experts shared their perspectives, experiences and knowledge of pre-identified key topics, and were able to raise additional issues they considered important. This article outlines the discussions of workshop participants on various policy areas, framed within the relevant scientific literature and policy documents. The results of this paper are intended to encourage researchers and policymakers to reflect on the policy challenges ahead and to enable the development of research agendas that investigate these areas and consider possible solutions. In particular, these challenges are crucial in the European research area, which is characterized by substantial policy divergence across institutes and countries, and limited direct funder intervention. Timely questions on the individual policy topics are outlined in Figure 1. The discussion addressed a range of subjects which were classified as follows: (1) multiplicity of platforms; (2) long-term sustainability of platforms; (3) lack of dedicated resources for data sharing; (4) access of commercial parties to medical data within platforms; (5) credit and recognition mechanisms in academia; and (6) the organization of data access committees within platforms.

### (1) Multiplicity of platforms

A plethora of platforms are currently being developed in parallel. Several consortia are undertaking distinctive projects within different research areas. Nevertheless, within some research domains multiple platforms are being set up. For example, as part of the Innovative Medicines Initiative (IMI), the platform under development by the BigData@Heart project and the European Health Data Evidence Network (EHDEN), could have overlapping functions with the euCanSHare platform. Therefore, the multiplicity of platforms was raised as a concern amongst workshop participants, specifically regarding platform competition, overlap, sustainability and potentially redundancy. Data sharing platforms may compete on separate levels – data deposition, data analysis and funding application – with each level subject to its own competitional dynamics. We outline these three levels in the following paragraphs.

***Data deposition*.** Platforms may compete to convince medical researchers to submit their cohort data. In order to allow for data interoperability in meta-analyses, the deposition of data on platforms require a certain degree of harmonization of variables, usually relying on a Common Data Model ^13, 14^. Consequently, the existence of multiple platforms within one research area might force data generators to choose between platforms, which is particularly relevant when data standards diverge. Multiple platforms within the same research domain adopting diverging standards could influence future data collection. For prospective harmonization, platforms themselves allow flexibility in designing research protocols, as the need for interoperability of data must not jeopardize the answering of principal research questions. Researchers are generally willing to harmonize data if possible, which is more complicated if standards between platforms diverge. However, the acceptance of different standards amongst platforms diminishes the chances of valuable information being left out partially or completely if harmonization with a data standard is not possible without significant data loss. One potential solution to combat the fragmentation of data over platforms would be to make data catalogues interoperable and allow them to provide information on data within other platforms. 

***Data analysis*.** Presently, various platforms aim to implement federated methods for analyses (e.g. through DataSHIELD, an infrastructure and series of R packages that enables the remote and non-disclosive analysis of sensitive research data) of datasets that cannot be transferred out of the institute ^15^. Federated methods adhere to the “code-to-data” principle instead of bringing data together centrally for analysis. In this way, these methods can help overcome legal restrictions and decrease patient concerns over data sharing, as data are not transferred elsewhere. Competition between platforms increases when they are located within the same research domain and offer overlapping services in terms of data quality or analysis tools.

***Funding application*.** Maintaining and improving platforms requires public funding or revenue streams. If the lifespan of a platform depends on public funding, then competition over project-based or structural funds might occur (see below).

Workshop participants argued that, despite potential competition, greater coordination and collaboration between platforms could be advantageous. The exchange of knowledge and expertise between platform hosts could facilitate addressing common problems and avoid unnecessary duplication of effort. Some initiatives already exist that encourage the exchange of ideas and solutions, such as the joint working groups of the EU-funded EUCAN program (of which euCanSHare is a member together with five other projects). Some participants proposed that the European Commission could take further action to enhance coordination between platforms, for example through coordination and support actions (CSA). The main objective of enhanced coordination should be to avoid creating a “jungle” of disconnected platforms that do not share expertise, compete directly with one another, and make it difficult for researchers to see the options available for data deposition and analysis.

Thus, the principal questions are (a) how competition and collaboration between platforms should ideally be managed; (b) how and when exchange of expertise should occur; and (c) which entities should take up coordinative action.

### (2) Long-term sustainability of data sharing platforms

In the past, maintenance of existing infrastructure was largely based on the repeated acquisition of project funding (usually 4–5 years), which is only suitable for time-limited projects with specific objectives ^16, 17^. In contrast, the development of data sharing platforms does not truly fit the criteria for project-based funding. First, platforms affect the ease of data sharing and the conduct of research *overall*. They contribute to and are used by a multitude of different research projects. Therefore, projects aimed at establishing platforms cannot be evaluated solely on the basis of traditional performance objectives (e.g. output articles) ^17^. Second, the valorization of platforms is *conditional* upon their long-term maintenance after initial development. As they only start adding value to research after the infrastructure has been fully developed, these projects cannot be expected to achieve their scientific objectives within the time limit of the initial project (i.e. the set-up phase). Some participants argued that in the past, the constant pursuit of entirely novel projects has led to an inability to sustain valuable infrastructures. From this perspective, an excessive dependency on short-term funding cycles can result in wasted investments, if the fruits of those investments are primarily born in the long-term. One recent review of projects using real-world data indicated that few ‘data source’ initiatives have sustainability plans for their output, and that many cease to exist after the initial term of the project ^18^. The investigators recommended that the “ *consideration of explicit mechanisms to ensure the sustainability of outputs by initiatives should be a priority and should be a requirement for any project proposal*” ^18^. While scientific literature on sustainability of data sharing platforms is relatively scarce, several policy reports describe potential sustainability mechanisms for data repositories.

The Organisation for Economic Co-operation and Development (OECD) report *Business Models for Sustainable Research Data Repositories* discusses potential revenue streams that would ensure long-term sustainability for data repositories ^16^. The report posits that there should be a shift from project-based funding to mixed-model funding. Mixed-model funding consists of both structural funding and cost-recovery mechanisms such as data deposition fees, data usage fees, data usage licenses, or charges for value-added services. These mechanisms could be applied in several ways to acquire funds. Allocating additional budget to data-generating research projects to pay data deposition fees could be a way for funders to promote platforms as the preferred providers for long-term data storage. Members of the Open Science unit at the Directorate-General for Research and Innovation have already put forward this proposal in general terms: *“The use of trusted or certified repositories and infrastructures like the European Open Science Cloud (EOSC) will be required for research data in some Horizon Europe work programs”* ^19^. In terms of mixed-model funding, another report from PwC EU Services puts forward recommendations along the same lines ^20^. Data infrastructures should shift focus towards data monetization and value-added services (recommendation [REC] #23), while mixed-business models should be explored to strike a “healthy balance” between public funding and other revenue streams (REC#25) ^20^.

Workshop participants warned that data deposition or usage fees could pose barriers that go against the spirit of Open Science. Similar arguments can be found in the literature, where concerns are raised that the use of fees or licenses for data usage could exacerbate existing inequalities between well-funded and less well-funded research groups, as the latter could be excluded from using platforms ^21^. Furthermore, funders might still end up paying for the fees themselves through various other routes (e.g. through funding allocated to individual research projects) ^21^. With regard to mixed-model funding, workshop participants noted that existing European infrastructures and repositories (e.g. the European Genome-phenome Archive [EGA], the Biobanking and Biomolecular Resources Research Infrastructure [BBMRI], Euro-Bioimaging) could theoretically support platforms, as they offer analytical and computational services that enhance reuse of data within these repositories. Hence, they could be considered a complementary computational component to supported data deposition services. As mentioned earlier, a greater dependence of platforms on this form of structural funding could lead to competition between platforms.

Policy documents and scientific literature also propose public-private partnerships as another way to ensure sustainability. Recently, the vision for the Health Research and Innovation Cloud (HRIC) was conceptualized as an overarching framework for existing data infrastructure, which promotes innovative funding mechanisms that involve private investors and partners to consolidate the outcomes of publicly-funded projects into long-term operational infrastructures ^22^. A European Commission CSA project (HealthyCloud) has been funded to prepare the design for these infrastructures, which also addresses their long-term sustainability. Likewise, the PwC report states that industry should establish partnerships or collaborations with data infrastructures and sponsor, fund or buy data and services (REC#26) ^20^. BBMRI’s Expert Centers, associated with the Biobanking and Biomolecular Resources Research Infrastructure – European Research Infrastructure Consortium (BBMRI-ERIC), could become one such novel public-private partnership model which brings together expertise from industry and the public for collaborative analysis in the pre-competitive research and development environment ^23^. Although public-private collaboration could be envisioned for data sharing platforms, access to medical data by commercial parties is an ethically sensitive issue, as elaborated upon later in this article. Furthermore, dependence on private partners for platform maintenance could lead to forms of *scope creep,* whereby platform design starts to cater to the goals of private partners rather than to the needs of academic researchers ^24, 25^.

Further reflection is needed on (a) how data sharing platforms can be made sustainable in the long-term; (b) how funding streams can be diversified and what consequences this would have for open science ideals; and (c) how to address ethical concerns over the access of commercial companies to medical data and the potential undue influence of commercial actors on platform design. Regardless of the specific funding model, it is imperative that future investments enable the expansion of infrastructures that already provide substantial value to research, rather than re-creating them from scratch with the renewal of each funding cycle. This strategy has already been successful in the set-up of Pan-European research infrastructures in specific domains (e.g. BBMRI for biobanks, European Clinical Research Infrastructure Network [ECRIN] for clinical trials) that build on a network of nationally supported hubs or nodes. 

### (3) Lack of dedicated resources for data sharing

Workshop participants voiced concerns over a lack of resources for data sharing. Many project-based studies might not possess sufficient funding to harmonize existing data resources before submitting them to a platform. Data harmonization requires local financial resources, manpower, time, and niche expertise, which may be an entry barrier for the deposition of data to platforms. It should be stressed that the process needs to be made affordable for smaller groups, and that inequities may emerge in terms of competitiveness if this problem is not addressed. This is particularly salient within a heterogenous research environment like the one in Europe, where divergence in research investments and academic cultures exists between different countries, and not all researchers have access to the same amount of funding. For example, if platform usage becomes more common, research teams which are unable to make data available to others could lose access to funding to sustain their cohorts. Whereas practitioners are well-aware of these challenges, policy reports do not yet take into account such inequities that could result from moving towards shared infrastructures for cohort data ^26^. For example, the report by PwC EU Services recommends making existing data FAIR on a demand-driven basis in order to maximize the return on investment for funders ^20^. As smaller teams might not possess a reliable system to share data, they might not be able to easily demonstrate actual demand for their data (e.g. based on various long-standing collaborations). Therefore, not all researchers may be able to acquire resources for harmonization. This problem occurs primarily after platform establishment, as data standards set by platforms might in the long-term influence primary data generation. This is because research teams might opt to produce data in compliance with existing data standards (i.e. prospective harmonization) to avoid the cost of complex retrospective harmonization at a later stage. Workshop participants also expressed concerns that research teams from particular areas (e.g. scientifically less competitive countries) might not be able to submit data due to a lack of resources, and that this might have downstream ethical consequences for the development of artificial intelligence (AI) algorithms. A lack of available data regarding populations of all geographical regions, socio-economic statuses and ethnicities diminishes the generalizability and fairness of algorithms. Consequently, the excluded populations would benefit less from the implementation of these tools in health care. 

Workshop participants expressed worries over a lack of institutional support for processing data sharing requests. While large infrastructure-like studies might have an elaborate administration to enable efficient processing of data requests, smaller, project-like studies might require principal investigators (PIs) to take on more administrative work ^27^. This creates barriers for data sharing, as teams must devote resources and time to data sharing that could be spent on their own research activities. Therefore, participants argued that a common infrastructure for data sharing would theoretically free up resources that were formerly dedicated to local systems. For this reason, the pooling of resources from larger projects could be one possible solution to address this barrier (e.g. by aiding with data harmonization). With regards to the governance infrastructure, Shabani *et al.* raised the argument that centralizing systems associated with individual Data Access Committees (DACs) makes resources available ^28^. They argue that smaller teams are better off setting up centralized systems as resources do not need to be invested in local systems, and centralized systems alleviate administrative burdens that come with sharing.

Key questions related to this theme include (a) how to fairly manage the transition costs for harmonizing data prior to platform submission for research teams; and (b) how the administrative burden (including legal considerations) for smaller research teams can be lowered so as to not interfere with their own research work.

### (4) Access to medical data by commercial parties

Data sharing platforms that contain harmonized and high-quality data can be of great value to commercial parties. For example, companies could use high-quality health data to train AI applications for clinical predictions, diagnostics, or allocating therapeutics ^29^. Some workshop participants argued that industry involvement or the creation of university spin-offs speeds up the translation of research results into tangible applications that can be put into practice. Others pointed out that the access of commercial companies to medical data remains an ethically sensitive topic.

Extensive empirical work, consisting of interview studies, focus groups and surveys finds that citizens generally dislike sharing data if it results in financial profit, showcasing only minor differences among patients and healthy populations. In any case, they want more information and control over secondary usage, especially if their data are entrusted to public organizations ^30– 35^. One recurring issue is that commercial parties are suspected to rank their commercial interests higher than the interests of patients as individuals or society. Perceptions of individual harm are related to privacy concerns, fear of stigmatization or the use of data against individuals, such as by private insurers or employers ^31, 32, 35– 37^. Collective harm might also be suspected if data access is seen as unjust, for example if there is no direct benefit for patients to donate data (i.e. lack of direct reciprocity) or if data access disproportionally furthers the profits of commercial entities (i.e. lack of indirect reciprocity) ^25, 35^. Additionally, unwarranted commodification of patient data can result in a loss of trust in public institutions, as the traditional roles of hospitals or universities are no longer fulfilled (i.e. the public good ethos is lost) ^31^. The public trusts that academic institutions, hospitals or biobanks live up to their expectations and uphold values that are consistent with their place in society. Behavior perceived to be contrary to these values can violate trust and undermine willingness to participate in research. Empirical work has shown that private research organizations, funding streams from private sources, connections between public researchers and pharmaceutical companies, public-private partnerships and the location of the data storage (national vs. international) all reduce trust and willingness to participate in medical health research ^25, 38^. Workshop participants also argued that existing concerns about data sharing are affected by (mis)trust in public institutions, such as the general reluctance to grant governmental access to health data. Some studies found that trust in the broader socio-political system (government, universities, industry, ethics committees and hospitals) is associated with willingness to contribute to biobanks ^39, 40^. Despite the stated concerns about commercial access to health data, patients may accept the existing tension within the status of commercial companies, who develop products indispensable to the advancement of public health, and yet operate on a for-profit basis ^25, 35, 36^. However, for-profit activities using public data could impact the motivation for sharing samples and data for research purposes ^25, 38, 41^. Altruism is currently considered the primary motivation for participation, and an increase in for-profit activities could corrode this motivation if data usage is not considered to sufficiently serve the public interest ^42, 43^. Instead, the desire for direct mutual benefit could replace altruism as the main motivator for research participation ^43^.

Various reports, based on consultations with UK citizens, indicate that there is a gray zone in terms of acceptable data sharing ^37, 44– 46^. Data sharing is acceptable with publicly funded institutions for uses that are in the public interest. Data sharing is inacceptable when organizations are private and uses are oriented towards the private interest. In-between these extremes, different levels of acceptability exist for uses which serve a mix of public and private benefit conducted by for-profit organizations in the health sector ^37, 46^. A heuristic for assessing acceptability includes the following topics: (a) the degree to which data reuse has a provable public benefit; (b) the orientation of the organization (public or private interest); (c) the level of anonymization of the data; and (d) rigor of safeguarding, access and storage protocols of data ^37^. Members of the UK public would generally accept a mix of public and private benefit, if the company operates within the health sector, and if the data are aggregated or anonymized ^37, 46^. Additionally, public partners should be involved in product development and a return of benefit should exist for the public partner (e.g. reduced prices of products) ^46^. Although they may initially feel skeptical about sharing data with commercial entities, a process of deliberation may inform and convince patients (particularly those that are unsure) to accept the involvement of commercial companies in developing data-driven services and products ^46^. This process of engagement and deliberation will become more important as the delineation between public and private research(ers) is blurring. In recent decades, there has been greater focus on translating basic research results into marketable products, with various forms of collaboration (e.g. public-private partnerships, university spin-offs) becoming increasingly prevalent.

The central questions about data sharing platforms are (a) whether access to medical data by commercial entities should take place; (b) which safeguards and governance mechanisms need to be installed for maintaining public trust; and (c) how an equitable distribution of benefits can be achieved when commercial companies are involved.

### (5) Credit and recognition mechanisms in academia

Workshop participants held diverging opinions regarding the need to alter recognition and evaluation systems. Some participants were very vocal about the need to thoroughly reform the reward system in academia, as they considered it detrimental to open science practices. Others were more skeptical of the ability of such reformations to incentivize data sharing, and instead stressed the lack of funding as the principal barrier. Within open science policy documents, such as the reports of the mutual knowledge exchange and expert/working groups of the European Commission, one central idea is that the design of credit and evaluation systems might prevent engaging in open science practices, including data sharing ^47– 56^. In the last decade, multiple manifestos have therefore called for substantial alterations to the academic reward system. Amongst these, the San Francisco Declaration on Research Assessment (DORA), the Leiden Manifesto for Research Metrics and The Metric Tide have all advocated moving away from commonly misused metrics, developing new so-called “responsible metrics” and reconsidering the relation between quantitative measures and qualitative assessment in research evaluation ^56– 58^.

Workshop attendees found that attributing due credit to those that generate and share valuable data is essential for these activities to maintain their value for researchers. Some participants considered the inclusion of data sharing as an authorship-worthy activity, favorable over the former situation where this was not in accordance with the guidelines of the International Committee of Medical Journal Editors (ICMJE). Researchers who use, or possibly misuse, data collected by others with whom they have no collaboration can be controversial these days, as exemplified in the editorial by Longo *et al.* who coined the term “research parasites” ^59^. However, an extrapolation of the collaborator model to data-intensive medical research leads to hyper-authorship and drives authorship inflation ^60, 61^. Opinions were divided on the adverse effects of hyper-authorship, with some participants expressing concerns over research integrity while others emphasized the necessity to credit those that share data. It was underlined that evaluation systems should not use the same criteria for academics who occupy different roles in the scientific pipeline. As the division of labor within science is constantly increasing, reward systems also need to integrate mechanisms to recognize specialized contributions to collaborative work. Mazumdar *et al.* underline the necessity of developing approaches to recognize those that engage routinely in collaborative science – “team scientists” – rather than lead their own projects ^62^. These approaches generally require less emphasis on author position and more focus on the nature of the reported contributions.

In recognition of the challenges to the authorship model, in 1997 Rennie *et al.* proposed a move away from authorship and to embrace contributorship ^63^. Over the years, several medical journals such as the Journal of the American Medical Association (JAMA), The New England Journal of Medicine (NEJM) and the British Medical Journal (BMJ) have adopted such contributor statements ^64^. In 2014, contributor statements were standardized through the Contributor Roles Taxonomy (CRediT), which has since become more popular ^65^. In the future, CRediT might further evolve by weighing relative contributions, being tailored to fit specific disciplines, being indexed with authorship metadata or being integrated upstream in the research pipeline ^65– 67^. One study found that medical researchers presently consider that author order still signals more valuable information for evaluation purposes, although future evolutions may make contributor statements more valuable ^68^. Participants of the workshop were generally positive about the evolution towards contributorship. Nevertheless, opinions diverged on the extent to which this shift needs to take place. While some argued that it is sufficient for journals to adopt contributor statements, others proposed a “film credits” model of contributorship, in which author order is completely abandoned. On this issue, participants judged that more reflection is necessary on (a) how individual contributions to collaborative science may best be portrayed; and (b) how these statements should be used for evaluation purposes.

Besides contributorship, several other initiatives exist that aim to attribute greater credit to the producing and sharing of datasets and digital objects more broadly. For example, within central repositories such as Zenodo and Figshare, usage and citations are being traced and aggregated centrally in the DataCite/Crossref Event Data service in standardized ways ^69, 70^. In a similar fashion, the Research Resource Identifier (RRID) enables key resources in biomedical literature to be cited using their identifiers such as antibodies, model organisms or software projects. Reuse and citation of openly available digital objects could then be understood as an indication of greater value in research, although many different contextual factors still need to be considered. Another example is the Bioresource Research Impact Factor (BRIF) which was developed to better recognize the value of datasets within biobanks ^71^. These initiatives illustrate that thought is being put into conferring value upon all research outputs (e.g. software, models, data) rather than solely on research articles.

In July 2019, the European Commission’s Expert Group on Indicators for Researcher’s Engagement with Open Science published the *Indicator Frameworks for Fostering Open Knowledge and Practices in Science and Scholarship* report. The report proposes the development of indicator toolboxes which, among others, entail infrastructure (or monitoring) indicators to gauge the evolution of open science practices at national, international or subject-specific levels ^56^. Workshop participants argued that data sharing platforms could, in principle, be designed to align with these goals by tracing usage of cohort data over *all* cohorts, which could then be centrally aggregated. The collation of such information via platforms could enable the development of detailed indicators and metrics for evaluation and analytical purposes, increase the oversight of research outcomes by funders, and inform science policy decisions ^72, 73^. They can also assist in better capturing the full social value of cohort data, such as usage that does not result in publication. Within academia more broadly, the implementation of such indicators and metrics for data objects are considered to foster data sharing practices. The discussion amongst workshop participants on their application to data sharing platforms focused on three key elements deemed essential to ensure the utility and validity of this kind of information: traceability, standardization, and coordination. Central questions are therefore: (a) can indicators relating to data sharing activities be traced within platforms?; (b) can this information be traced in a meaningful and standardized fashion?; and (c) can the fragmentation of standards across platforms be avoided? Future discussions should aim to explore technical possibilities of collecting indicators on data sharing through platforms and the value of these indicators within scientific domains.

### (6) Data access committees (DACs)

Individual-level patient data of population or disease cohorts constitute privacy-sensitive personal data and are subject to controlled-access models. DACs exist to manage these processes, and they may be associated with institutions, biobanks, consortia or with individual study teams ^74– 76^. These DACs assess the legitimacy of the proposed research project and whether the researcher is *bona fide* (i.e. affiliated with a scientific institution, competency) ^77^. DACs are composed of persons with scientific and ethico-legal expertise. Some health consortia use a decentralized model to govern secondary access to health data, in which each participating cohort has its own DAC linked to the research team or institution. In that case, by adding cohorts to platforms, additional DACs are added which operate *in parallel* to each other. In contrast, other consortia may also employ centralized models to govern data access, in which one DAC linked to the consortium decides upon data access for requests to all studies. Some workshop participants argued that, depending on the degree of (de)centralization of access procedures, the total administrative burden and the burden for individual cohorts may differ.

Workshop participants raised that, if cohorts maintain fully decentralized data access procedures, requesting access for multiple datasets might cause longer waiting times. For example, when the submitted proposals are challenged on scientific grounds, modifications to proposals might require several rounds of recirculation to all committees involved, each of which has their own schedule for processing those requests. Furthermore, DACs comment on the scientific validity of the proposal in uncoordinated fashion, meaning that applicants might receive contradictory or conflicting comments (e.g. on the preferred method of analysis), or individual DACs might simply not respond. Since the DACs linked to each requested dataset are immediately involved in the evaluation of scientific proposals, there is also redundancy in access evaluation and the total bureaucratic load increases ^78^. The bureaucratic burden for individual DACs could also increase, particularly when the number of data requests rises substantially or if many initial proposals for data use are not scientifically sound ^78^. Furthermore, it is possible that DACs could adhere to different standards to evaluate proposals ^28, 78^. One DAC might constitute a board which conducts an elaborate assessment by looking into the scientific validity of the proposal or the skills of the applicant, while another has a single scientist decide upon access to datasets ^28^. Some workshop participants suggested that platforms should be non-interventionist towards the organization of DACs, comparable to the policy followed by the European Genome-phenome Archive (EGA). Others stressed the need for DACs to operate efficiently and considered that decentralized models create unnecessary redundancy. Under the latter view, several different solutions may be pursued.

One proposed model was to use layered models of DACs whereby some tasks are centrally performed (e.g. assessing the scientific validity of proposals) while others are left to individual institutions (e.g. assessing consent and legal requirements). A similar concept is employed within the Monica Risks, Genetics, Archiving and Monograph (MORGAM) project. Adopting such a model within the platform would mean that when entering data into the platform, research teams are given the option either to establish an additional DAC in parallel to the others, or to join existing partially centralized DACs that perform specified tasks.

Another proposed model was instating a formal data access structure called METADAC (Managing Ethico-social, Technical and Administrative issues in Data Access). A METADAC used to manage access to multi-type data and samples of seven longitudinal cohort studies in the UK. Data requests are evaluated using specific criteria, aimed at embodying three key principles: independence and transparency, interdisciplinarity, and participant-centric decision-making. The independence of the evaluation procedure combats data “hugging” or hoarding by individuals who are internal to the study ^79^. In a joint response, funders of scientific research in the UK have already endorsed the necessity for independent data access procedures, and claimed to be committed to address instances *“in which usage of data is only permitted in collaboration with a study team”*. The particularities of a study, such as the availability and relevance of data, weaknesses and limitations of study design and data, can be addressed by representation of principal investigators within the METADAC (e.g. as observers or active participants) ^79^.

Fully centralized DACs can ensure that the data of contributing cohorts can be used interoperably for a common purpose. The International Cancer Genome Consortium (ICGC) uses such a singular DAC composed of independent experts to oversee access to all of its data ^74^. The experiences with data access in the ICGC indicate that the aforementioned problems regarding the inefficiency of processing requests and guaranteeing that access evaluation is independent from personal interests of data producers are generally absent ^80^. Nevertheless, the use of centralized DACs requires greater standardization of consent elements, the governance model and oversight practices. Such standardization efforts could be resisted by participating cohorts, who are concerned about losing control over data usage ^28^.

Technical solutions exist to alleviate the bureaucratic burden of verifying the compatibility of access requests with consent provisions. Working Groups of the Global Alliance for Genomics and Health (GA4GH) developed the Data Usage Ontology (DUO) and the Automatable Discovery and Access Matrix (ADA-M), which aim to turn consent information into machine-readable codes that can be matched with incoming data access requests ^81, 82^. This matching can take place by using smart contracts within blockchain technology for sharing medical data ^83^. These technologies semi-automate the matching of consent criteria and therefore could reduce the administrative burden of having to manually check consent for each proposal. It is also valuable in situations where consent documents were collected in different languages, as understandable codes can be made visible to researchers that wish to request data. 

Salient issues on DACs include (a) the viability of different models within platforms and their compatibility with semi-automating parts of the data access request system and (b) the degree of independence DACs require. In the future, these models will need to be assessed for their ability to streamline administrative procedures and their scalability, while addressing possible concerns of PIs about an asserted loss of control over data usage.

## Conclusion

This work on barriers, challenges and opportunities for data sharing platforms is the result of discussions with experts within the “euCanSHare Workshop on Incentives for Data Sharing” and the reviewing of existing scientific literature and policy documents. The future research agenda should further investigate the topics outlined here within the context of data sharing platforms. Data sharing platforms face multiple challenges related to their multiplicity and long-term sustainability, their perceived and actual data security, access to data by commercial parties, lack of resources to prepare data for sharing, sharing of academic credit and recognition, and the management of data access. A critical reflection and a thorough discussion is necessary to create a suitable policy environment in which data sharing platforms can thrive. Platforms by themselves should not be considered a panacea that solves *all* problems of data sharing in health research. Rather, they are to be understood as technical instruments that need to be undergirded by sound science policy. In this way, unexpected pitfalls for data sharing can be mitigated. Assessing public opinion on public-private collaboration and the access of commercial partners to medical data will be essential to obtain a social license for greater industry involvement. Comprehensive solutions to these pertinent policy questions will enable platforms to establish themselves as core components of a productive academic ecosystem. When these conditions are met, future investments can be directed towards building on the foundations laid by others and expanding the platforms for sustainable scientific benefit. This mode of working can best be encapsulated by revisiting (in part) the words of Descartes on the scientific method: *“The best minds would be led to contribute to further progress, each one according to their bent and ability* (…) *so that one man might begin where another left off; and thus, in the combined lifetimes and labors of many, much more progress would be made by all together than anyone could make by themselves.*”

## Data availability

The workshop was not a research study. Therefore, no ethics approval from an IRB was required or obtained for conducting this workshop, in which the terms and conditions for data sharing could be disclosed and evaluated. Audio files of the workshop were retained only for drafting an initial version of the manuscript. In addition, they were made temporarily available to participants via Google Drive. No verbatim transcription of statements of individuals took place. At the beginning of the workshop, a verbal agreement was reached with participants to use audio files only for purposes of this publication. The audio recordings also cannot be reasonably anonymized. Audio-files therefore cannot be disclosed because they will be destroyed.

## Author contributions

Conceptualization: TD

Visualization: TD

Writing – Original Draft Preparation: TD

Writing – Review & Editing: CA, FWA, AB, RC, MGF, JLG, MRJ, KK, KL, MTM, VP, GP, SEP, COS, JSM, SS, MS, GV, DSV, PB

Project Administration: TD, PB, MS, KL

Supervision: PB, MS, KL
