# Supplementary material for: An agenda-setting paper on data sharing platforms: euCanSHare workshop
Source: Open Res Eur. 2021 Nov 23;1:80. Originally published 2021 Jul 19. [Version 2] doi: 10.12688/openreseurope.13860.2 (PMC10445835; doi:10.12688/openreseurope.13860.2)
Supplement: Peer review history

# Peer review: Reviewer response for version 1

**DOI:** 10.21956/openreseurope.14943.r27631

**Published:** 2021-10-25

**Authors:** Bernice Elger

**Affiliations:** 1 Institute for Biomedical Ethics, University of Basel, Basel, Switzerland

The article provides an adequate and detailed overview of the ethical, regulatory and practical issues involved in the use and setting up of data sharing repositories. For many of the described issues, an overarching question is whether to use a centralized approach versus competition of platforms, as it has been explained in detail for example concerning data access commissions.

 The aim of such platforms is to make data available to others through open access (OA). Interestingly, this concept is not as such questioned as long as appropriate consent of patients or those whose data are used has been obtained. Indeed, OA of data seems to be required and automatically judged as “good” in current discussions. Here we see only one example of issues that will require further thought in the future and of many yet unresolved details.

Is the argument persuasive and supported by appropriate evidence?

Yes

Is the work clearly and cogently presented?

Yes

Is the topic of the essay discussed accurately in the context of the current literature?

Yes

Does the essay contribute to the cultural, historical, social understanding of the field?

Yes

Reviewer Expertise:

NA

I confirm that I have read this submission and believe that I have an appropriate level of expertise to confirm that it is of an acceptable scientific standard.

# Response

**Published:** 2021-11-22

**Authors:** Thijs Devriendt

**Affiliations:** KU Leuven, Belgium

Our general position is that making data FAIR ( Findable, Accessible, Interoperable and Reusable) is good because it serves the public interest. The primary objective of academia is to produce valid knowledge that is (in medical research) responsive to the health needs of the population. Furthermore, the allocation of public funding to this endeavour also implies that funds should be spent in an efficient and responsible way to maximize the return on investment for society. Making data FAIR helps the production of knowledge and maximize the return on investment by (a) facilitating meta-analyses, reanalyses, and the exploration of secondary hypotheses in data; (b) enabling data-inductive methods for analysis (e.g., machine learning); and (c) preventing redundancy in data collection. In cases where data sharing could lead to the production of “false” knowledge because, for instance, the risk of misinterpretation exists, it is advisable that data producers are actively involved in the analysis. Nevertheless, one may argue that despite the risk of misinterpretation, the overall benefits of openly sharing (i.e., without active involvement of data partners) still outweigh the downsides. There are still various factors that still need to be respected for data sharing to be “good”. First, the persons whose data is being used must have given their informed consent to sharing, or sharing data should be grounded in a legitimate or public interest. Second, the purposes of data sharing must not conflict with the interests or values of individual research participants or with important societal (or communal) values. This means that data sharing for particular purposes may not be desirable (e.g., data sharing for purposes that may cause harm to communities). Thirdly, data sharing should take into account power differentials in between partners in data sharing. This means that, although the *purpose* of data sharing may be good, the *process* of data sharing is also relevant. One such example is data sharing between researchers in high-income countries and low-middle income countries. In this case, efforts to establish an equitable partnership may include knowledge exchange to compensate for structural inequalities. We have not further elaborated on the cases in which data sharing is undesirable in the article.

# Peer review: Reviewer response for version 1

**DOI:** 10.21956/openreseurope.14943.r27628

**Published:** 2021-09-20

**Authors:** Wendy Lipworth

**Affiliations:** 1 Sydney Health Ethics, Faculty of Medicine and Health, The University of Sydney, Sydney, NSW, Australia

Thank you for the opportunity to review this article, which describes the "data sharing agenda" that emerged from a euCanSHare workshop. In this article, the authors articulate several areas of concern for which solutions are needed and distill each issue down to a set of policy/research questions that need to be answered. 

 As a bioethicist, I was struck by the degree to which each of these issues and questions is morally charged. This is true even of apparently "technical" or "logistical" issues (e.g. platform overlap, funding, data access committees models). For example (as the authors note) inequities can be introduced by adopting particular approaches to harmonising data, particular funding models or models for membership of data access committees. 

 The article does a good job of articulating the ethical dimensions of each issue. Both (brief) theoretical arguments and empirical studies are drawn upon to try to shed light on how some of these issues might be conceptualised and addressed (particularly in relation to commercial access and academic credit).

 While these high-level summaries of the ethical issues are appropriate for the purposes of this article, they only hint at the complexity of each issue. I, therefore, think that more thought needs to be given (if not in this article then as a next step) to how to manage this moral complexity. In particular, thought should be given to 

1. precisely *how* "[t]he future research agenda should further investigate the topics outlined here within the context of data sharing" and 
2. where research needs to be replaced (or at least supplemented) with ongoing reflexivity and multi-stakeholder deliberation about whatever data models are put into place. 

The principles of "responsible (research and) innovation" might be useful here, as these recognise the need for both anticipation of ethical issues and ongoing revision of whatever decisions are made.

 There are a few minor grammatical errors in the article

- p5/12: "output articles" should be "output of articles"
- p7/12: "inacceptable" should be "unacceptable"

Is the argument persuasive and supported by appropriate evidence?

Yes

Is the work clearly and cogently presented?

Yes

Is the topic of the essay discussed accurately in the context of the current literature?

Yes

Does the essay contribute to the cultural, historical, social understanding of the field?

Yes

Reviewer Expertise:

Bioethics

I confirm that I have read this submission and believe that I have an appropriate level of expertise to confirm that it is of an acceptable scientific standard.
